# No Evidence for a Functional Role of Bi-Directional Notch Signaling during Angiogenesis

**DOI:** 10.1371/journal.pone.0053074

**Published:** 2012-12-28

**Authors:** Sven S. Liebler, Anja Feldner, M. Gordian Adam, Thomas Korff, Hellmut G. Augustin, Andreas Fischer

**Affiliations:** 1 Vascular Signaling and Cancer (A270), German Cancer Research Center Heidelberg, (DKFZ-ZMBH Alliance), Heidelberg, Germany; 2 Department of Vascular Biology and Tumor Angiogenesis (CBTM), Medical Faculty Mannheim, Heidelberg University, Mannheim, Germany; 3 Institute of Physiology and Pathophysiology, Division of Cardiovascular Physiology, University of Heidelberg, Heidelberg, Germany; 4 Division of Vascular Oncology and Metastasis (A190), German Cancer Research Center Heidelberg (DKFZ-ZMBH Alliance), Heidelberg, Germany; University of Maastricht (UM), The Netherlands

## Abstract

The Delta-Notch pathway is a signal exchanger between adjacent cells to regulate numerous differentiation steps during embryonic development. Blood vessel formation by sprouting angiogenesis requires high expression of the Notch ligand DLL4 in the leading tip cell, while Notch receptors in the trailing stalk cells are activated by DLL4 to achieve strong Notch signaling activity. Upon ligand binding, Notch receptors are cleaved by ADAM proteases and gamma-secretase. This releases the intracellular Notch domain that acts as a transcription factor. There is evidence that also Notch ligands (DLL1, DLL4, JAG1, JAG2) are processed upon receptor binding to influence transcription in the ligand-expressing cell. Thus, the existence of bi-directional Delta-Notch signaling has been proposed. We report here that the Notch ligands DLL1 and JAG1 are processed in endothelial cells in a gamma-secretase-dependent manner and that the intracellular ligand domains accumulate in the cell nucleus. Overexpression of JAG1 intracellular domain (ICD) as well as DLL1-ICD, DLL4-ICD and NOTCH1-ICD inhibited endothelial proliferation. Whereas NOTCH1-ICD strongly repressed endothelial migration and sprouting angiogenesis, JAG1-ICD, DLL1-ICD and DLL4-ICD had no significant effects. Consistently, global gene expression patterns were only marginally affected by the processed Notch ligands. In addition to its effects as a transcription factor, NOTCH1-ICD promotes cell adhesion to the extracellular matrix in a transcription-independent manner. However, JAG1-ICD, DLL1-ICD and DLL4-ICD did not influence endothelial cell adhesion. In summary, reverse signaling of Notch ligands appears to be dispensable for angiogenesis in cellular systems.

## Introduction

Notch signaling is a highly conserved signal transmitter between adjacent cells to regulate many cellular differentiation steps. Notch receptors (NOTCH1-NOTCH4 in mammalians) are single-pass transmembrane proteins that bind to ligands of the Delta, Serrate and Lag-2 (DSL) families. If the ligand exerts a “pulling force” [1], the conformational changes induce cleavage events to release the extracellular Notch domain by ADAM proteases (ADAM-10) and the intracellular Notch domain (ICD) via gamma-secretase. Intriguingly, NOTCH-ICD acts as a transcription factor that once released from the plasma membrane, enters the nucleus and interacts with several transcriptional activators to modulate global gene expression patterns [2].

In addition to this canonical signaling, cleaved Notch intracellular domains influence other signaling cascades, in particular Wnt signaling, independently of transcriptional control (non-canonical Notch signaling) [3]. As another example, NOTCH1-ICD promotes conformational changes of β1-integrins via R-Ras to promote integrin-mediated cell adhesion to extracellular matrix proteins [4].

Within the vasculature, Notch signaling is essential to counterbalance vascular endothelial growth factor (VEGF) during the outgrowth of novel blood vessels (angiogenesis) [5]. One critical mechanism during angiogenesis is the selection of tip cells, which guide new vessel sprouts and stalk cells that stay behind and form the vessel lumen. Initially, several endothelial cells respond to VEGF and acquire tip cell characteristics. However, VEGF induces expression of the Notch ligand DLL4 that subsequently activates Notch receptors on neighboring cells. In turn, Notch receptors rapidly induce the transcriptional repressors HEY1 and HEY2 [6], which down-regulate expression of the VEGF receptors (VEGFR2 and VEGFR3). Thus, an individual tip cell (strong DLL4 expression, little Notch activity) is rapidly selected. This tip cell signals via DLL4 to adjacent cells, which acquire stalk cell characteristics (strong Notch activity, low VEGF signaling). Consistently, Notch gain-of-function results in a poorly branched vascular network, while Notch loss-of-function causes excessive proliferation of endothelial cells which form a dense, tip cell rich, and chaotic vascular network that is poorly functional [2], [5].

It is tempting to speculate that Notch ligands do not only activate Notch receptors (forward signaling) but also transmit signals to the nucleus of the ligand-expressing cell (reverse signaling). Hughes and colleagues proposed such a function in the vasculature [7]. Bi-directional signaling of transmembrane ligand-receptor protein pairs is known, e.g. from the ephrin ligand/Eph receptor system, which is needed for vascular patterning [8]. Given this scenario, DLL4 would not only induce the stalk cell phenotype in adjacent cells but also promote the tip cell phenotype in the DLL4-expressing cell. There is increasing evidence from non-vascular cell types that such bi-directional Notch signaling does indeed occur.

Several groups have reported that Notch ligands of the Delta (DLL1, DLL3, DLL4 in humans) and Jagged/Serrate (JAG1, JAG2 in humans) families are cleaved upon binding of Notch receptors. Processing of the ligands appears to be highly similar to that of the receptors [9]. The Delta protein in Drosophila is cleaved by Kuzbanian, a metalloprotease related to mammalian ADAM10, to release the extracellular domain [10], [11]. Mammalian Notch ligands (DLL1, JAG2) also undergo cleavage by ADAM proteases [12]–[16]. The remaining membrane-bound protein is - similar to Notch receptors - cleaved by the gamma-secretase complex to release the intracellular domain [13], [14], [17], [18]. Ligand cleavage is dependent on Notch receptor binding [19], and the intracellular domains of the ligands locate – at least partially – to the nucleus [13], [20], [21].

A potential role of ligand-ICDs within the cell nucleus is unclear. There is some evidence that JAG1-ICD activates gene expression in vitro [13], [14], [19]. Additionally, the overexpression of Serrate (Jagged)-ICD inhibits primary neurogenesis in Xenopus embryos [22]. The intracellular domain of DLL1 (DLL1-ICD) is enriched in the nucleus of neural stem cells and specifically binds SMAD transcription factors (SMAD2, SMAD3, SMAD4) to enhance TGF-β/Activin signaling. The overexpression of DLL1-ICD induces neuron formation in P19 embryonic carcinoma cells [23], but it was also reported that DLL1-ICD induces growth arrest by inducing expression of the cell cycle inhibitor p21 in several cell types [24]. Recently, a different mode of action for ligand-ICDs was proposed. Instead of regulating transcription on their own, JAG1-ICD may disrupt the formation of the NOTCH1-ICD nuclear complex with RBPJκ and MAML1 in HEK293 cells [25]. This implies a mechanism that inhibits Notch forward signaling. This may particularly be important for such cells that simultaneously receive and send Notch signals.

Based on these data we hypothesized that bi-directional Notch signaling should occur in endothelial cells to fine-tune angiogenesis. However, our data indicate that even the forced expression of processed ligands in endothelial cells exerts little effects on cellular functions and global gene expression patterns.

## Results and Discussion

**Figure 1:**
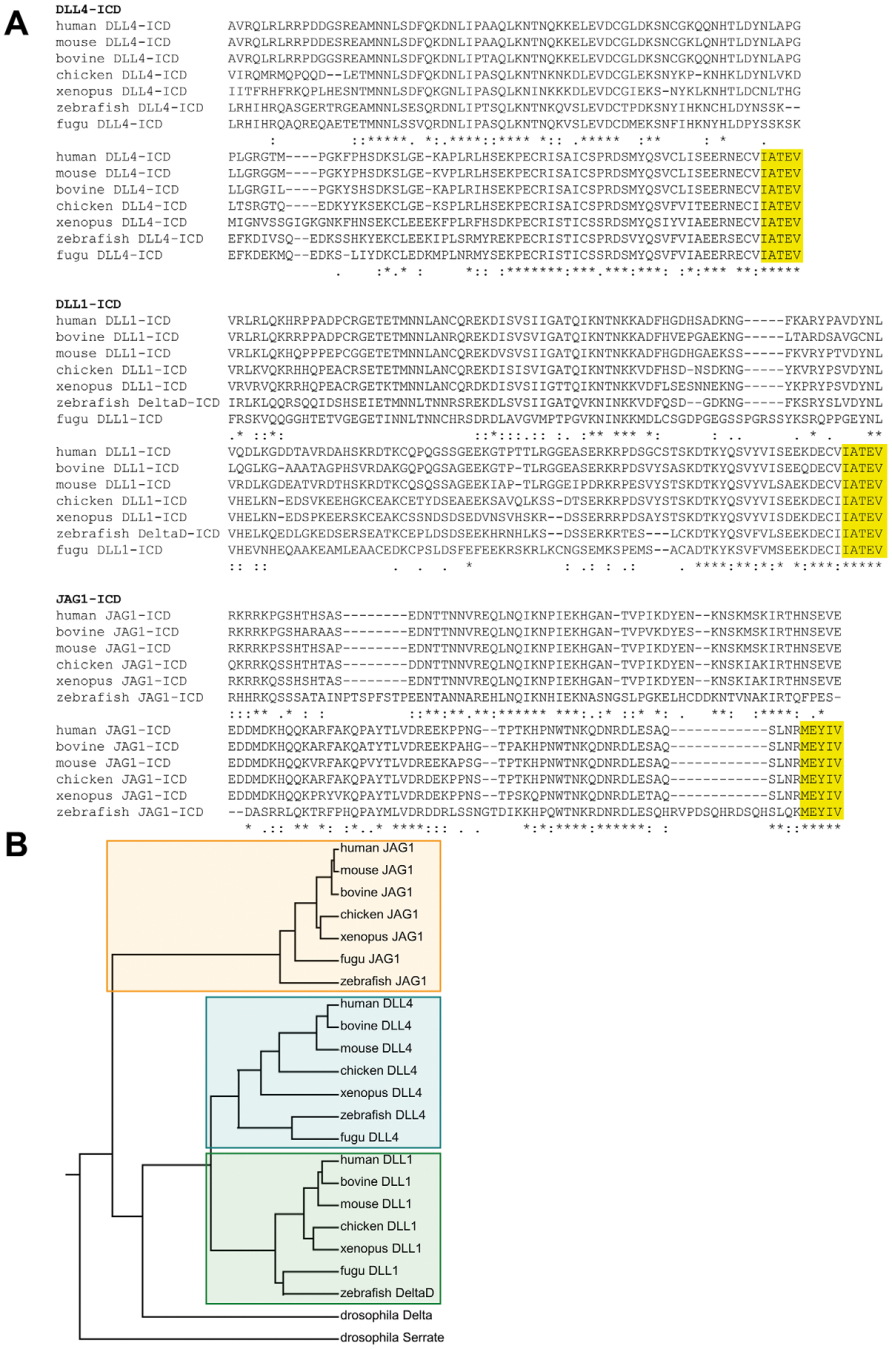
Intracellular domains of DLL4, DLL1 and JAG1 are highly conserved. (A) ClustalW alignment of the intracellular domains of human DLL4, DLL1, and JAG1 with their respective homologues from mouse, cow, chicken, xenopus, zebrafish, and fugu revealed a high grade of conservation between all vertebrate species. The carboxyterminal PDZ motif (highlighted in yellow) is 100% conserved in all cases. (B) A dendrogram from a ClustalW alignment of the full-length proteins from vertebrates and drosophila proves that the proteins as a whole are also highly conserved between species. Colored boxes show the closer relationship of DLL1 and DLL4 compared to JAG1.

The intracellular domains of the Notch ligands DLL1, DLL4 and JAG1 are highly conserved and contain a carboxyterminal PDZ binding motif (**Figure 1**). Popovic et al., have reported that the intracellular protein fragment does not adopt a higher ordered structure in aqueous solution, but this may chance upon protein interactions [26], [27]. Interestingly, the PDZ binding motif of Delta ligands (IATEV) differs from that of Jagged/Serrate ligands (MEYIV) indicating that these ligands also recruit different intracellular proteins. Indeed, it was reported that JAG1 binds to the Ras-binding protein afadin (AF6) in a PDZ-dependent manner [19], [28], whereas DLL1 and DLL4 interact with the PDZ domains of MAGI1, MAGI2 [29], [30] and DLG1 [31].

### JAG1 is Processed by Gamma-secretase in Endothelial Cells

**Figure 2:**
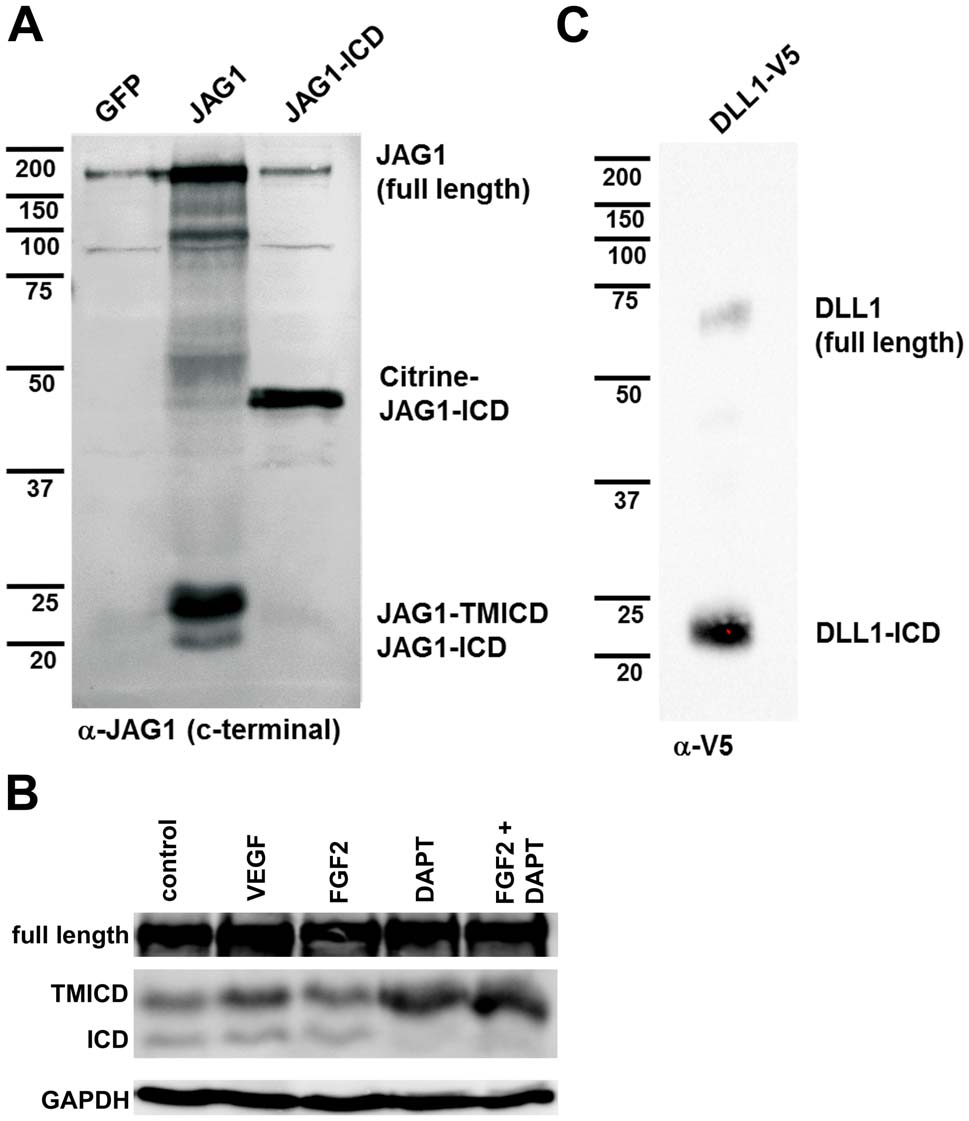
JAG1 is processed by gamma-secretase. HUVEC were transduced with adenoviruses expressing GFP, full length JAG1. The intracellular JAG1 domain fused to Citrine, or DLL1 tagged with V5 at the carboxyterminus. (A) JAG1 was processed and the cleavage products have the predicted size of the transmembrane-intracellular domain fragment (TM-ICD) and the free intracellular domain (ICD). The membrane was blotted with JAG1 antibodies recognizing the carboxyterminal intracellular domain. (B) Treatment with VEGF or FGF2 did not significantly increase ligand cleavage compared to solvent as control. Inhibition of gamma-secretase activity with 25 µM DAPT prevented the formation of the intracellular domain and led to accumulation of the uncleaved TM-ICD fragment. (C) The predicted size of the DLL1 intracellular domain, tagged with V5, was detected in HUVEC lysates by Western blotting.

Processing of Notch ligands by ADAM proteases and gamma-secretase, in particular JAG1, has been observed in several non-vascular cell lines and primary cells [10]–[25]. We determined if JAG1 becomes processed in primary human endothelial cells of umbilical veins (HUVEC). Cells were transduced with adenovirus to enhance the expression of full-length human JAG1 protein, which is only weakly expressed in venous cells. Western blotting with an antibody recognizing the intracellular JAG1 domain revealed the presence of not only full length protein, but also fragments with the proposed molecular weights of the transmembrane-intracellular JAG1 fragment (TM-ICD) and of the intracellular domain (ICD) (**Fig. 2a**). Addition of pro-angiogenic endothelial growth factors had no effect on JAG1 processing itself (**Fig. 2b**). Thus, we could detect the same JAG1 protein cleavage pattern in endothelial cells as seen with Notch receptors before.

**Figure 3:**
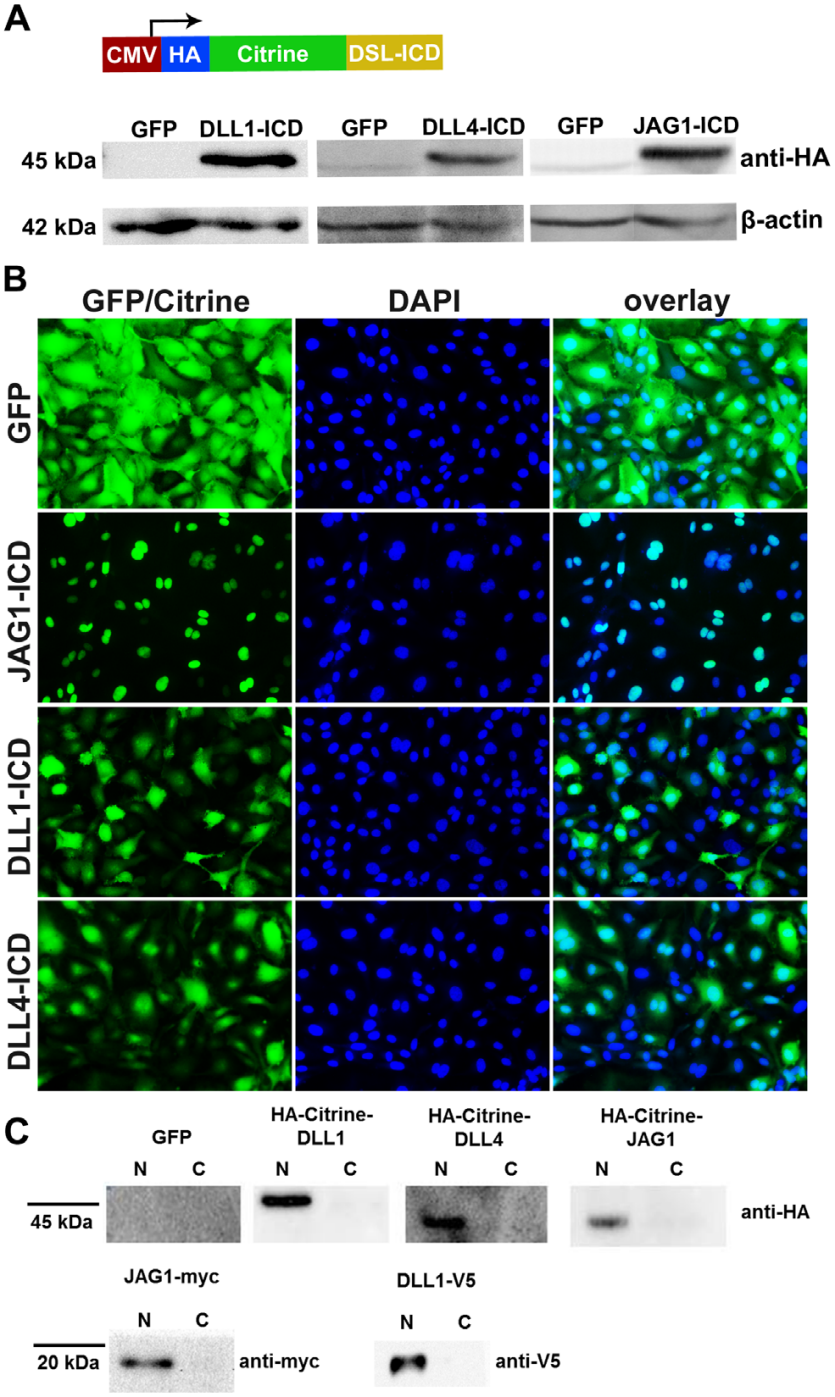
Localisation of Notch ligand intracellular domains. (A) Scheme of the Notch ligand (DSL) intracellular domain (ICD) expression cassette in adenoviral vectors. Western blotting revealed expression of the fusion proteins in total lysates. (B) JAG1-ICD was localized predominantly in the nucleus, whereas DLL1-ICD and DLL4-ICD proteins were also localized in the cytoplasm. Scale bar, 100 µm. (C) Ligand-ICDs fused with Citrine were detected only in nuclear fractions (N) but not in the cytoplasmic fractions (C). HUVEC expressing full-length JAG1 or DLL1 fused to a c-terminal myc or V5-tag were lysed to obtain nuclear and cytoplasmic fractions. The processed intracellular fragments were detected only in the nuclear extracts by Western blotting.

The critical step of Notch receptor activation after ligand binding is cleavage by the gamma-secretase complex. We treated endothelial cells with a widely used gamma-secretase inhibitor (25 µM DAPT) to verify that endogenous gamma-secretase activity is responsible for JAG1 cleavage. The gamma-secretase inhibitor fully prevented the formation of JAG1-ICD. As a consequence, the protein levels of the TM-ICD fragment were increased (**Fig. 2b**). The lack of suitable antibodies precluded further analysis of the Delta ligands (DLL1, DLL4). However, expression of DLL1 fused to a V5 tag at the carboxyterminus was also processed and the V5-tagged intracellular fragment could be detected in cell lysates (**Fig. 2C**). Moreover, the processed intracellular domains of JAG1 and DLL4 were highly enriched in the nuclear but not cytoplasmic cell fraction (**Fig. 3c**). In summary, the data indicate that not only Notch receptor but also ligand processing occurs in endothelial cells and requires gamma-secretase activity. Thus, blockage with typical “Notch inhibitors” like DAPT should be interpreted more carefully and one should account that also the Notch ligands may be affected.

### Notch Ligand-ICDs Inhibit Endothelial Proliferation

The Notch ligands JAG1, DLL1 and DLL4 are essential for several critical endothelial cell functions in vertebrates. We overexpressed processed Notch ligand intracellular domain fragments in human primary endothelial cells (HUVEC) by adenoviral transduction to reveal potential functions of putative Notch reverse signaling. The short ligand-ICDs were fused to the GFP variant Citrine to achieve more stable protein expression (**Fig. 3a**). The aminotermial fusion led the PDZ motif intact. Citrine-Delta-ICDs (DLL1-ICD and DLL4-ICD) were observed within the cytoplasm and nucleus by fluorescent microscopy, while Citrine-JAG1-ICD location was almost exclusively detected in the nucleus (**Fig. 3b**). Additionally, we detected strong accumulation of these proteins in nuclear cell fractions, but not in cytoplasmic ones **(** **Fig. 3c** **)**.

**Figure 4:**
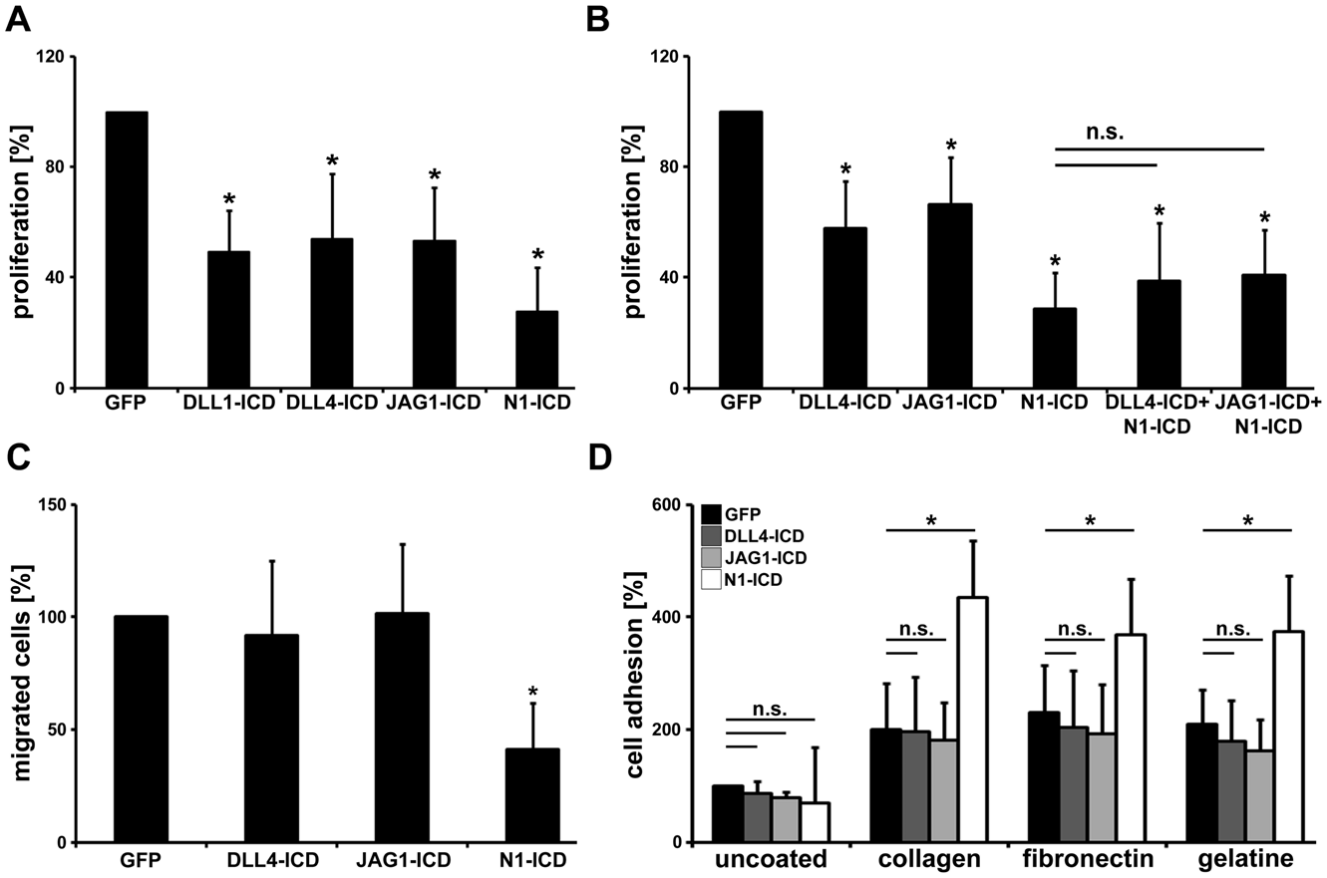
Notch ligand intracellular domains inhibit cellular proliferation but not migration and adhesion. (A,B) Adenoviral expression of Delta and Jagged intracellular domains (ICD) in HUVEC inhibited BrdU incorporation indicating decreased cell proliferation. NOTCH1-ICD had stronger repression activity that was not altered by co-expression of DLL4-ICD or JAG1-ICD. n = 3 independent experiments. (C) Chemotactic migration of HUVEC through a transwell filter was not altered by ligand-ICDs but strongly impaired by NOTCH1-ICD expression. n = 3 independent experiments. (D) Adhesion of HUVEC to extracellular matrix proteins was enhanced by NOTCH1-ICD but not by ligand-ICDs. n = 3 independent experiments. Data are presented as mean +SD. *, p<0.05.

Notch receptor signaling regulates endothelial cell proliferation, adhesion, migration and sprouting angiogenesis. This has been shown by others and us, e.g. by forced expression of constitutively active NOTCH1 intracellular domain (NOTCH1-ICD) [2], [7], [32], [33]. Using the same experimental set-up [32], we analyzed how JAG1-ICD, DLL1-ICD and DLL4-ICD expression affects these critical cellular functions. NOTCH1-ICD expression served as a control to confirm classical Notch forward signaling. First, we determined endothelial cell proliferation by measuring BrdU incorporation into HUVEC. This revealed significantly reduced cell proliferation by approximately 45–50% due to expression of processed Notch ligands DLL1-ICD, DLL4-ICD and JAG1-ICD. NOTCH1-ICD expression inhibited endothelial proliferation by approximately 70% (**Fig. 4a**). This finding is in line with a previous report showing that DLL1-ICD blocks proliferation of several cell types including HUVEC [24].

It was recently shown that JAG1-ICD is able to interact with NOTCH1-ICD and to disrupt the formation of the NOTCH1-ICD-RBPJκ-Mastermind complex in a non-vascular cell line (HEK293) [25]. However, co-expression of ligand-ICDs (DLL4-ICD or JAG1-ICD) with NOTCH1-ICD in HUVEC did not alter the proliferation block induced by NOTCH1-ICD (**Fig. 4b**).

### No Effects of Ligand-ICDs on Migration and Adhesion

Secondly, we analyzed the effects of processed Notch ligands on chemotactic cell migration towards a VEGF gradient in a trans-well chamber. The number of migrated endothelial cells was significantly reduced once the cells expressed the active intracellular domain of NOTCH1 as shown before [32]. In contrast, expression of the intracellular domains of the ligands had no effects on chemotactic cell migration (**Fig. 4c**).

NOTCH1-ICD is able to activate β1-integrins in a non-transcriptional manner and this promotes endothelial cell adhesion to the extracellular matrix protein collagen-I [4]. An endothelial cell adhesion assay was performed to analyze such a potential function of ligand-ICDs. While NOTCH1-ICD significantly enhanced adhesion of HUVEC to cell culture dishes coated with the β1-integrin substrates collagen, fibronectin or gelatin, expression of DLL4-ICD or JAG1-ICD did not alter endothelial cell adhesion (**Fig. 4d**).

### No Effects of Processed Notch Ligands on Sprouting Angiogenesis

**Figure 5:**
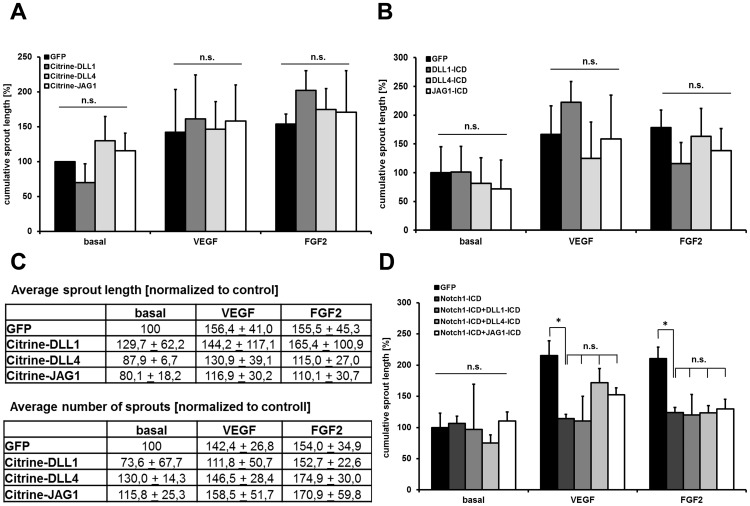
Notch ligand ICDs do not affect sprouting angiogenesis. NOTCH1-ICD or ligand-ICD expressing HUVEC were cultured as spheroids in collagen and treated with 25 ng/ml VEGF, FGF-2 or PBS as control. The cumulative length of all sprouts was measured. Data are presented as mean cumulative sprout length per spheroid +SD. n = 3 independent experiments with 10 spheroids per condition. (A,B) Expression of Citrine-tagged ligand-ICDs or such without this tag did not significantly alter sprouting behavior. (C) The average sprout length was slightly but not significantly (p>0.05 in all cases) reduced after ligand-ICD expression. The average number of capillary sprouts was not altered. (D) NOTCH1-ICD expression prevents VEGF and FGF2-induced sprouting angiogenesis. However, this was not significantly affected by co-expression of ligand-ICDs. *, p<0.05. n.s., not significant.

Cultured endothelial cells faithfully mimic several aspects of sprouting angiogenesis when suspended in collagen as spheroids. It is well known that activation of Notch signaling inhibits or even abolishes endothelial sprouting in this assay as well as in vivo (e.g. the postnatal mouse retina or during formation of the intersomitic vessels in zebrafish embryos) [2], [7]. Consequently, expression of NOTCH1-ICD in HUVEC spheroids prevented VEGF or FGF2-induced sprouting angiogenesis in collagen beds **(** **Fig. 5d** **)** as shown before [32]. However, expression of ligand-ICDs had no consistent and significant effects on endothelial sprouting (**Fig. 5a,b**). Neither sprout numbers nor sprout length was altered (**Fig. 5c**). To exclude that the Citrine tag interferes with ligand-ICD functions, we overexpressed these proteins without a tag but again this did not cause significant alterations during sprouting angiogenesis (**Fig. 5b**). Since JAG1-ICD was shown to interfere with the Notch nuclear complex [25], we tested whether coexpression of ligand-ICD could interfere with the functions of NOTCH1-ICD. However, JAG1-ICD, DLL1-ICD and DLL4-ICD had no significant effects on NOTCH1ICD-mediated inhibition of sprouting angiogenesis (**Fig. 5d**). Thus, it is very unlikely that Notch reverse signaling affects critical steps of angiogenesis at least in well established cellular systems.

### Notch Ligand-ICDs have Little Effects on Global Gene Expression

**Figure 6:**
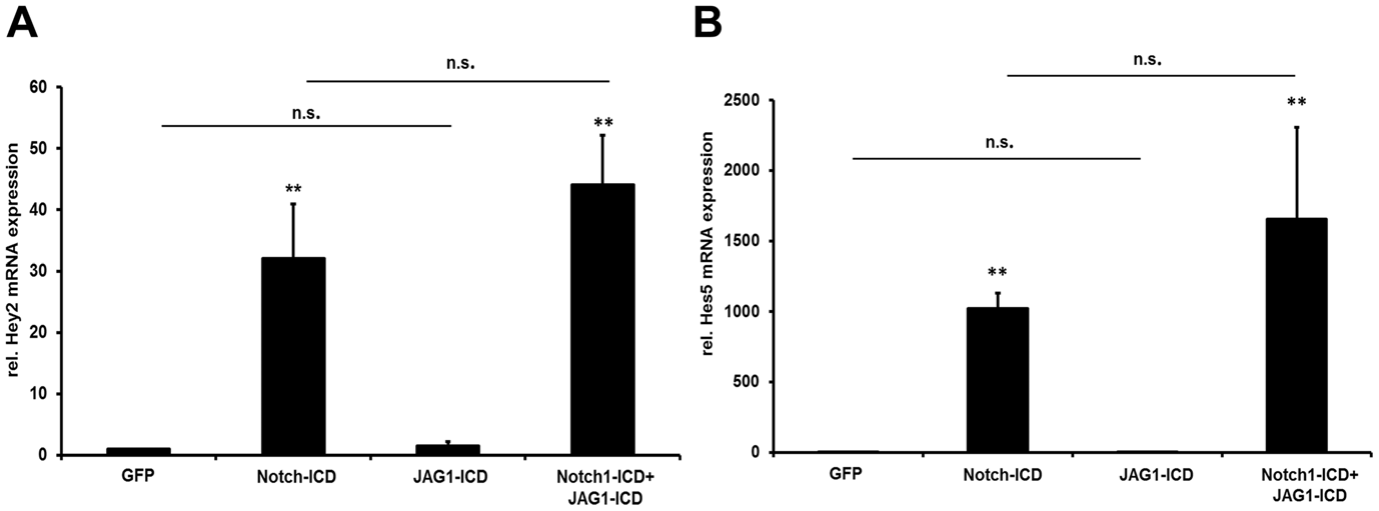
JAG1-ICD does not interfere with NOTCH1-ICD in target gene induction. HUVEC express only little amounts of HEY2 and HES5 mRNA. (A, B) Expression of NOTCH1-ICD induces strong expression of these genes 48 h after adenoviral transduction. This was not significantly affected by co-expression of JAG1, which itself had also no significant effects on mRNA expression of these endothelial Notch target genes. **, p<0.01. n = 3 independent experiments with 3 technical replicates.

Studies in non-vascular cell types proposed a function of ligand-ICDs on transcriptional control, based on luciferase reporter assays or mRNA detection of single genes [13], [14], [19], [23]–[25]. Although ligand-ICD expression had little effects on typical endothelial cell functions we tested mRNA expression levels of several candidate genes involved in angiogenesis that are regulated by Notch signaling. Quantitative real-time PCR revealed no alterations of the classical Notch target genes HEY1, HEY2 and DLL4, as well as no constant changes of mRNA expression of VEGF receptors (VEGFR1, VEGFR2, VEGFR3) 36 hours after transduction with DLL4-ICD or JAG1-ICD expressing adenovirus (data not shown). Moreover, co-expression of JAG1-ICD did not significantly interfere with the strong induction of HEY2 and HES5 expression by NOTCH1-ICD **(** **Fig. 6** **)**.

Additionally, we performed unbiased whole transcriptome screening to detect alterations in gene expression patterns after adenoviral ligand-ICD expression in HUVEC. Expression of DLL1-ICD, DLL4-ICD and JAG1-ICD had only marginal effects on global gene expression patterns. After applying stringent filtering, only very few transcripts (DLL1-ICD: 12 transcripts, DLL4-ICD: 1 transcript, JAG1-ICD: 7 transcripts) were significantly regulated more than two-fold compared to GFP expression (**table 1**). Remarkably, PCDH17 was induced by all three ligand-ICDs, but the function of this non-clustered protocadherin is poorly understood [34]. In contrast, NOTCH1-ICD significantly regulated almost 300 transcripts in the same experiment as already described [32].

**Table 1:** Transcripts regulated by Notch ligand intracellular domains.

	transcript	fold change	description	
**DLL1-ICD regulated**				
	PCDH17	3,3	protocadherin 17	
	GJA5	3,2	gap junction protein, alpha 5, 40kDa	
	VIPR1	2,7	vasoactive intestinal peptide receptor 1	
	GDF3	2,5	growth differentiation factor 3 (GDF3), mRNA.	
	SELL	2,5	selectin L (lymphocyte adhesion molecule 1)	
	LRRC17	2,4	leucine rich repeat containing 17	
	*unknown*	2,4	cDNA: FLJ21027 fis, clone CAE07110	
	HIST1H2BD	2,4	histone cluster 1, H2bd	
	IL33	2,3	interleukin 33	
	IL1RAPL1	2,2	interleukin 1 receptor accessory protein-like 1	
	E2F2	−2,5	E2F transcription factor 2 (E2F2)	
	DNMT1	−2,9	DNA (cytosine-5-)-methyltransferase 1	
**DLL4-ICD regulated**				
	PCDH17	2,2	protocadherin 17	
				
**JAG1-ICD regulated**				
	PCDH17	2,2	protocadherin 17	
	GJA5	2,2	gap junction protein, alpha 5, 40kDa	
	PPFIBP2	2,1	PTPRF interacting protein, binding protein 2 (liprin beta 2)	
	VIPR1	2,1	vasoactive intestinal peptide receptor 1	
	C18orf34	1,7	chromosome 18 open reading frame 34	
	STS-1	−2,2	Cbl-interacting protein Sts-1	
	CENTG3	−2,3	centaurin, gamma 3	

**Table 1** **.** mRNA transcripts significantly changed 36 h after adenoviral infection of HUVEC with DLL1-ICD, DLL4-ICD or JAG1-ICD. Adenoviral GFP expression served as control.

In summary, our data suggest that Notch ligands are proteolytically processed in a highly similar manner as the Notch receptors. Although proliferation was significantly reduced after forced expression of Notch ligand-ICDs, there was no significant net effect on sprouting angiogenesis in vitro, hence the relevance of ligand intracellular domains in endothelial cells remained unclear. If Notch ligand-ICDs indeed signal to the nucleus one would expect to observe more alterations in endothelial cell behavior and gene expression patterns after overexpression of the intracellular ligand domains. Since this was not the case we have to revise our hypothesis. It may instead be that the most important aspect of ligand processing is to limit the protein amount of ligands after receptor binding or to terminate signaling. However, it is extremely difficult to address this issue, as any mutations within the ligand itself will most likely affect their receptor activation capability. In this way, studies employing ligands lacking the intracellular domain, the PDZ binding motif or the cleavage sites should cautiously be interpreted.

## Materials and Methods

### Plasmids and Viruses

Full length human JAG1 cDNA with a c-terminal myc tag in the pCMV-ENTR vector was from OriGene (RC10516) and shuttled by EcoRI-EcoRV into pENTR3c. The intracellular domains of murine Dll1, Dll4 and Jag1 were PCR amplified and ligated into pCS2p and pCS2-HA-Citrine, which leads to expression of fusion proteins with the HA tag and the fluorescent Citrine at the aminoterminal site. The ligand-ICD without tag and the Citrine-ligand-ICD cassettes were released with HindIII (fill in)-EcoRI and transferred into pENTR3c (DraI-EcoRI). The adenoviral NOTCH1-ICD construct was described before [32]. It encodes the aminoterminally HA-tagged ICD of murine Notch1 followed by an IRES-EGFP cassette. All cDNA inserts in the pENTR3c vectors were shuttled into the adenoviral pAd-V5-DEST vector by Gateway cloning (Invitrogen). Adenoviruses were generated in HEK293 cells using the ViraPower adenoviral Expression Systems kit (Invitrogen). Semiconfluent endothelial cells were transduced with multiplicity of infection of 50.

### Cell Culture

Primary human endothelial cells from umbilical veins (HUVEC) were purchased from PromoCell and cultured in endothelial growth medium (ECGM2) with supplements (PromoCell) and 10% FCS (Biochrom). Only cells of passages 2–6 were used for experiments.

### Endothelial Cell Proliferation, Migration, Adhesion and Sprouting

Cell proliferation was measured by the incorporation of 5-bromodeoxyuridine (BrdU). HUVEC were seeded into in 96-well plates (10,000 cells/well) 24 h after viral transduction. BrdU was added (24 h later) for 12 h and DNA incorporation was quantified by ELISA (BrdU Cell Proliferation ELISA, Roche).

Chemotactic cell migration was determined in a modified Boyden chamber. The filter (8 µm pores) was coated with collagen-I. HUVEC (48 h after transduction) were seeded on top of the membrane and 25 ng/ml VEGF or FGF-2 was added to the lower chamber as a chemoattractant. After 4 h incubation the cells at the lower side of the filter were fixed with EtOH, stained by Giemsa solution and counted.

Cell adhesion was detected 48 h after viral infection. Cells (20,000 in 50 µl per well) were seeded in 96-well plates. The plates were either untreated or coated with collagen-I (3 mg/ml), 0.2% gelatin or fibronectin (20 µg/ml; Sigma-Aldrich). The plates were incubated for 30 min and then shaken for 10s in a standardized procedure. After washing with medium, the adherent cells were fixed with 4% paraformaldehyde, washed and stained with crystal violet (5 mg/ml in 2% EtOH) for 10 min. Cells were washed again with water, dried and lysed with 2% SDS. Adhesion was quantified by measuring the absorption of the solution at 550 nm.

The endothelial spheroid sprouting angiogenesis assay was performed as described [32], [35]. In short, HUVEC were suspended in 20% methocel (Sigma-Aldrich) and cultured as hanging drops overnight. Thereby, cell spheroids (400 cells each) formed. The spheroids were washed and embedded in collagen. Medium lacking growth factors (basal medium) or containing VEGF or FGF-2 (final concentration 25 ng/ml) was added to the collagen beds. Capillary-like structures were assessed 24 h later. Ten spheroids per condition were analyzed and the assay was repeated three times with different HUVEC samples. Shown is the average cumulative length of all sprouts per spheroid.

### Gene Expression Profiling

Total RNA was isolated from cell cultures using the RNeasy kit (Qiagen) and cDNA was generated with the SuperScript II Reverse Transcriptase kit (Invitrogen) using random hexamer primers. 1 µl of 1∶5 diluted cDNA was used for real-time quantitative PCR using the POWER SYBR Green Master Mix in a 25 µl reaction on an ABI StepOnePlus cycler (Applied Biosystems). Normalization was done with the house-keeping genes HPRT1 and OAZ1.

Whole transcriptomic gene expression profiling was performed by the DKFZ Genomics Core Facility as described before [32]. HUVEC (passage 2) were adenovirally infected in quadruplicates and 36 h later total RNA was harvested. One well was used to verify the effects on cell proliferation, while total RNA was harvested from the other three wells. Remaining DNA was digested with DNase I. RNA quality was determined on an Agilent 2100 Bioanalyzer. Screening on Illumina Sentrix human WG-6 v3.0 bead chips was performed as described. Raw data were quantile normalized and analyzed by Bead Studio 3.1.3 software with the Genome Studio Plugin (Illumina). Transcripts with detection p-values smaller 0.05 were selected. Expression changes were calculated with the Illumina Custom error model including a Benjamini and Hochberg algorithm for multi-testing corrections. Probes with p-values smaller 0.001 were filtered. The complete data set of the NOTCH1-ICD vs. GFP experiment can be found in the NCBI GEO database (GSE18035).

### Western Blotting

Cells were lysed in RIPA buffer containing proteinase inhibitors (Roche) and 1 mM DTT. Nuclear and cytoplasmic extracts were prepared according to the Dignam & Roeder protocol [36]. Protein samples were separated by SDS-PAGE and transferred to nitrocellulose filters (Whatman). Membranes were blocked with 5% skim milk in PBS and incubated with primary antibodies at 4°C overnight. After washing and incubation with secondary peroxidase-coupled antibodies for 1 h at room temperature, detection was achieved with AceGlow substrate (Peqlab). Primary antibodies: JAG1 (C-20, Santa Cruz), GAPDH (ab9483, Abcam), β-actin (sc-1616, Santa Cruz), anti-HA tag (F7; Santa Cruz).

### Statistical Analyses

Results are expressed as means plus/minus standard deviations. Comparisons between groups were done by a 2-sided t-test. p-values smaller 0.05 were considered significant.

### ClustalW Alignment

The ClustalW alignment was performed with an online software tool (http://www.genome.jp/tools-bin/clustalw). The intracellular domains of vertebrate DLL1, DLL4, and JAG1were defined by similarity to the known human and murine ICD sequences (www.uniprot.org). Ensembl protein sequences (www.ensembl.org) used for the alignment: human DLL1 ENSP00000355718, mouse DLL1 ENSMUSP00000014917, bovine DLL1 ENSBTAP00000042093, chicken DLL1 ENSGALP00000018210, xenopus DLL1 ENSXETP00000048762, zebrafish DeltaD ENSDARP00000089996, fugu DLL1 ENSTRUP00000026316, drosophila Delta FBpp0083153, human DLL4 ENSP00000249749, mouse DLL4 ENSMUSP00000099575, bovine DLL4 ENSBTAP00000013680, chicken DLL4 ENSGALP00000013851, xenopus DLL4 ENSXETP00000046677, zebrafish DLL4 ENSDARP00000094097, fugu DLL4 ENSTRUP00000038560, human JAG1 ENSP00000254958, mouse JAG1 ENSMUSP00000028735, bovine JAG1 ENSBTAP00000009631, chicken JAG1 ENSGALP00000038500, xenopus JAG1 ENSXETP00000004994, zebrafish JAG1 ENSDARP00000121170, fugu JAG1 ENSTRUP00000003565, drosophila Serrate FBpp0084498.
